# Supplementary material for: Computational methods to simulate molten salt thermophysical properties
Source: Commun Chem. 2022 Jun 2;5:69. doi: 10.1038/s42004-022-00684-6 (PMC9814384; doi:10.1038/s42004-022-00684-6)
Supplement: Supplementary file 1 — Supplementary Data 1 [file 42004_2022_684_MOESM1_ESM.docx]

**Supplementary Data 1**

Talmage Porter^1,‡^, Michael Vaka^1,‡^, Parker Steenblik^1^,Dennis Della Corte^1,*^

**Table S1 of Flibe Structural Properties**

| Pair | First Peak Position Angstrom | Temperature K | Composition (LiF-BeF­_2_) | Method | Source |
| --- | --- | --- | --- | --- | --- |
| Be-F | 1.567 | 0 | 66.6-33.3 | AIMD | Baral, K., San, S., Sakidja, R., Couet, A., Sridharan, K., & Ching, W.-Y. (2021). Temperature-dependent properties of molten Li2BeF4 Salt using Ab initio molecular dynamics. ACS Omega, 6(30), 19822-19835. |
|  | 1.58 | 673.15 | 50-50 | Experiment X-ray diffraction | Vaslow, F., & Narten, A. H. (1973). Diffraction pattern and structure of molten BeF2–LiF solutions. The Journal of Chemical Physics, 59(9), 4949-4954. https://doi.org/10.1063/1.1680711 |
|  | 1.58 | 828.15 | 66.6-33.3 | Experiment X-ray diffraction | Vaslow, F., & Narten, A. H. (1973). Diffraction pattern and structure of molten BeF2–LiF solutions. The Journal of Chemical Physics, 59(9), 4949-4954. https://doi.org/10.1063/1.1680714 |
|  | 1.575 | 850 | 66.6-33.3 | AIMD | Baral, K., San, S., Sakidja, R., Couet, A., Sridharan, K., & Ching, W.-Y. (2021). Temperature-dependent properties of molten Li2BeF4 Salt using Ab initio molecular dynamics. ACS Omega, 6(30), 19822-19835. |
|  | 1.58 | 873 | 66.6-33.3 | Modified RIM | Salanne, M., Simon, C., Turq, P., Heaton, R. J., & Madden, P. A. (2006). A First-Principles Description of Liquid BeF2 and Its Mixtures with LiF:  2. Network Formation in LiF−BeF2. *The Journal of Physical Chemistry B*, *110*(23), 11461-11467. https://doi.org/10.1021/jp061002u |
|  | 1.55 | 973 | 66.6-33.3 | AIMD | Nam, H. O., Bengtson, A., Vörtler, K., Saha, S., Sakidja, R., & Morgan, D. (2014). First-principles molecular dynamics modeling of the molten fluoride salt with Cr solute. Journal of Nuclear Materials, 449(1-3), 148-157. https://doi.org/10.1016/j.jnucmat.2014.03.015 |
|  | 1.5 | 973 | 66.6-33.3 | BPNN | Lam, S. T., Li, Q.-J., Ballinger, R., Forsberg, C., & Li, J. (2021). Modeling LiF and FLiBe Molten Salts with Robust Neural Network Interatomic Potential. *ACS Applied Materials & Interfaces*, *13*(21), 24582-24592. https://doi.org/10.1021/acsami.1c00604 |
|  | 1.56 | 973 | 66.6-33.3 | DPMD | Rodriguez, A., Lam, S., & Hu, M. (2021). Thermodynamic and Transport Properties of LiF and FLiBe Molten Salts with Deep Learning Potentials. ACS Applied Materials & Interfaces, 13(46), 55367-55379. https://doi.org/10.1021/acsami.1c17942 |
|  | 1.575 | 1000 | 66.6-33.3 | AIMD | Baral, K., San, S., Sakidja, R., Couet, A., Sridharan, K., & Ching, W.-Y. (2021). Temperature-dependent properties of molten Li2BeF4 Salt using Ab initio molecular dynamics. ACS Omega, 6(30), 19822-19835. |
|  | 1.58 | 1018.15 | 80-20 | Experiment X-ray diffraction | Vaslow, F., & Narten, A. H. (1973). Diffraction pattern and structure of molten BeF2–LiF solutions. The Journal of Chemical Physics, 59(9), 4949-4954. https://doi.org/10.1063/1.1680720 |
|  | 1.58 | 1023.15 | 66.6-33.3 | Experiment X-ray diffraction | Vaslow, F., & Narten, A. H. (1973). Diffraction pattern and structure of molten BeF2–LiF solutions. The Journal of Chemical Physics, 59(9), 4949-4954. https://doi.org/10.1063/1.1680717 |
|  | 1.57 | 1135 | 50-50 | PIM | Smith, A. L., Capelli, E., Konings, R. J. M., & Gheribi, A. E. (2020). A new approach for coupled modelling of the structural and thermo-physical properties of molten salts. Case of a polymeric liquid LiF-BeF2. *Journal of Molecular Liquids*, *299*, 112165. https://doi.org/https://doi.org/10.1016/j.molliq.2019.112166 |
| F-F | 2.572 | 0 | 66.6-33.3 | AIMD | Baral, K., San, S., Sakidja, R., Couet, A., Sridharan, K., & Ching, W.-Y. (2021). Temperature-dependent properties of molten Li2BeF4 Salt using Ab initio molecular dynamics. ACS Omega, 6(30), 19822-19835. |
|  | 2.563 | 673.15 | 50-50 | Experiment X-ray diffraction | Vaslow, F., & Narten, A. H. (1973). Diffraction pattern and structure of molten BeF2–LiF solutions. The Journal of Chemical Physics, 59(9), 4949-4954. https://doi.org/10.1063/1.1680712 |
|  | 2.563 | 828.15 | 66.6-33.3 | Experiment X-ray diffraction | Vaslow, F., & Narten, A. H. (1973). Diffraction pattern and structure of molten BeF2–LiF solutions. The Journal of Chemical Physics, 59(9), 4949-4954. https://doi.org/10.1063/1.1680715 |
|  | 2.588 | 850 | 66.6-33.3 | AIMD | Baral, K., San, S., Sakidja, R., Couet, A., Sridharan, K., & Ching, W.-Y. (2021). Temperature-dependent properties of molten Li2BeF4 Salt using Ab initio molecular dynamics. ACS Omega, 6(30), 19822-19835. |
|  | 2.61 | 873 | 66.6-33.3 | Modified RIM | Salanne, M., Simon, C., Turq, P., Heaton, R. J., & Madden, P. A. (2006). A First-Principles Description of Liquid BeF2 and Its Mixtures with LiF:  2. Network Formation in LiF−BeF2. *The Journal of Physical Chemistry B*, *110*(23), 11461-11467. https://doi.org/10.1021/jp061002u |
|  | 2.5 | 973 | 66.6-33.3 | BPNN | Lam, S. T., Li, Q.-J., Ballinger, R., Forsberg, C., & Li, J. (2021). Modeling LiF and FLiBe Molten Salts with Robust Neural Network Interatomic Potential. *ACS Applied Materials & Interfaces*, *13*(21), 24582-24592. https://doi.org/10.1021/acsami.1c00606 |
|  | 2.58 | 973 | 66.6-33.3 | DPMD | Rodriguez, A., Lam, S., & Hu, M. (2021). Thermodynamic and Transport Properties of LiF and FLiBe Molten Salts with Deep Learning Potentials. ACS Applied Materials & Interfaces, 13(46), 55367-55379. https://doi.org/10.1021/acsami.1c17944 |
|  | 2.58 | 973 | 66.6-33.3 | AIMD | Nam, H. O., Bengtson, A., Vörtler, K., Saha, S., Sakidja, R., & Morgan, D. (2014). First-principles molecular dynamics modeling of the molten fluoride salt with Cr solute. Journal of Nuclear Materials, 449(1-3), 148-157. https://doi.org/10.1016/j.jnucmat.2014.03.016 |
|  | 2.588 | 1000 | 66.6-33.3 | AIMD | Baral, K., San, S., Sakidja, R., Couet, A., Sridharan, K., & Ching, W.-Y. (2021). Temperature-dependent properties of molten Li2BeF4 Salt using Ab initio molecular dynamics. ACS Omega, 6(30), 19822-19835. |
|  | 2.56 | 1018.15 | 80-20 | Experiment X-ray diffraction | Vaslow, F., & Narten, A. H. (1973). Diffraction pattern and structure of molten BeF2–LiF solutions. The Journal of Chemical Physics, 59(9), 4949-4954. https://doi.org/10.1063/1.1680721 |
|  | 2.563 | 1023.15 | 66.6-33.3 | Experiment X-ray diffraction | Vaslow, F., & Narten, A. H. (1973). Diffraction pattern and structure of molten BeF2–LiF solutions. The Journal of Chemical Physics, 59(9), 4949-4954. https://doi.org/10.1063/1.1680718 |
| F-Li | 1.887 | 0 | 66.6-33.3 | AIMD | Baral, K., San, S., Sakidja, R., Couet, A., Sridharan, K., & Ching, W.-Y. (2021). Temperature-dependent properties of molten Li2BeF4 Salt using Ab initio molecular dynamics. ACS Omega, 6(30), 19822-19835. |
|  | 1.85 | 673.15 | 50-50 | Experiment X-ray diffraction | Vaslow, F., & Narten, A. H. (1973). Diffraction pattern and structure of molten BeF2–LiF solutions. The Journal of Chemical Physics, 59(9), 4949-4954. https://doi.org/10.1063/1.1680713 |
|  | 1.85 | 828.15 | 66.6-33.3 | Experiment X-ray diffraction | Vaslow, F., & Narten, A. H. (1973). Diffraction pattern and structure of molten BeF2–LiF solutions. The Journal of Chemical Physics, 59(9), 4949-4954. https://doi.org/10.1063/1.1680716 |
|  | 1.893 | 850 | 66.6-33.3 | AIMD | Baral, K., San, S., Sakidja, R., Couet, A., Sridharan, K., & Ching, W.-Y. (2021). Temperature-dependent properties of molten Li2BeF4 Salt using Ab initio molecular dynamics. ACS Omega, 6(30), 19822-19835. |
|  | 1.81 | 873 | 66.6-33.3 | Modified RIM | Salanne, M., Simon, C., Turq, P., Heaton, R. J., & Madden, P. A. (2006). A First-Principles Description of Liquid BeF2 and Its Mixtures with LiF:  2. Network Formation in LiF−BeF2. *The Journal of Physical Chemistry B*, *110*(23), 11461-11467. https://doi.org/10.1021/jp061002u |
|  | 1.8 | 973 | 66.6-33.3 | BPNN | Lam, S. T., Li, Q.-J., Ballinger, R., Forsberg, C., & Li, J. (2021). Modeling LiF and FLiBe Molten Salts with Robust Neural Network Interatomic Potential. *ACS Applied Materials & Interfaces*, *13*(21), 24582-24592. https://doi.org/10.1021/acsami.1c00605 |
|  | 1.88 | 973 | 66.6-33.3 | DPMD | Rodriguez, A., Lam, S., & Hu, M. (2021). Thermodynamic and Transport Properties of LiF and FLiBe Molten Salts with Deep Learning Potentials. ACS Applied Materials & Interfaces, 13(46), 55367-55379. https://doi.org/10.1021/acsami.1c17943 |
|  | 1.88 | 973 | 66.6-33.3 | AIMD | Nam, H. O., Bengtson, A., Vörtler, K., Saha, S., Sakidja, R., & Morgan, D. (2014). First-principles molecular dynamics modeling of the molten fluoride salt with Cr solute. Journal of Nuclear Materials, 449(1-3), 148-157. https://doi.org/10.1016/j.jnucmat.2014.03.014 |
|  | 1.893 | 1000 | 66.6-33.3 | AIMD | Baral, K., San, S., Sakidja, R., Couet, A., Sridharan, K., & Ching, W.-Y. (2021). Temperature-dependent properties of molten Li2BeF4 Salt using Ab initio molecular dynamics. ACS Omega, 6(30), 19822-19835. |
|  | 1.85 | 1018.15 | 80-20 | Experiment X-ray diffraction | Vaslow, F., & Narten, A. H. (1973). Diffraction pattern and structure of molten BeF2–LiF solutions. The Journal of Chemical Physics, 59(9), 4949-4954. https://doi.org/10.1063/1.1680722 |
|  | 1.85 | 1023.15 | 66.6-33.3 | Experiment X-ray diffraction | Vaslow, F., & Narten, A. H. (1973). Diffraction pattern and structure of molten BeF2–LiF solutions. The Journal of Chemical Physics, 59(9), 4949-4954. https://doi.org/10.1063/1.1680719 |
