# Supplementary material for: Computational methods to simulate molten salt thermophysical properties
Source: Commun Chem. 2022 Jun 2;5:69. doi: 10.1038/s42004-022-00684-6 (PMC9814384; doi:10.1038/s42004-022-00684-6)
Supplement: Supplementary file 2 — Description of Additional Supplementary Files [file 42004_2022_684_MOESM2_ESM.pdf]

Description of Additional Supplementary Files

File Name: Supplementary Data 1

Description: Table of Flibe structural properties, broken down by atomic pairs as experimentally measured or computationally predicted at various temperatures.
